# Confocal laser scanning microscopy as a valuable tool in Diptera larval morphology studies

**DOI:** 10.1007/s00436-014-4125-0

**Published:** 2014-09-19

**Authors:** Andrzej Grzywacz, Tomasz Góral, Krzysztof Szpila, Martin J. R. Hall

**Affiliations:** 1 Chair of Ecology and Biogeography, Faculty of Ecology and Environment Protection, Nicolaus Copernicus University, Lwowska 1, 87-100 Toruń, Poland; 2 Imaging and Analysis Centre, Natural History Museum, London, UK; 3 Department of Life Sciences, Natural History Museum, London, UK

**Keywords:** Diptera, Cyclorrhapha, Larva, Morphology, Confocal laser scanning microscope, Clearing technique

## Abstract

Larval morphology of flies is traditionally studied using light microscopy, yet in the case of fine structures compound light microscopy is limited due to problems of resolution, illumination and depth of field, not allowing for precise recognition of sclerites’ edges and interactions. Using larval instars of cyclorrhaphan Diptera, we show the usefulness of confocal laser scanning microscopy (CLSM) for studying the morphological characters of immature stages by taking advantage of the autofluorescent properties of cephaloskeleton structures. We compare data obtained from killed but unprepared larvae with those from larvae prepared by clearing according to two commonly used methods, either with potassium hydroxide or with Hoyer’s medium. We also evaluated the CLSM application for examining already slide-mounted larvae stored in museum collections and those freshly prepared. Our results indicate that CLSM and 3D reconstruction are excellent for visualizing small, compound structures of cylrorrhaphan larvae cephaloskeleton, if appropriate clearing techniques, i.e. the application of KOH, are used. Maximum intensity projection of confocal data sets obtained from material freshly prepared and that stored in museum collection does not differ. Because of this and the fact that KOH is commonly used as a clearing method to examine the cephaloskeleton of Diptera larvae, it is possible, and highly recommended, to use slides already prepared with this method for re-examination by CLSM. We conclude that CLSM application can be an invaluable source of data for studies of larval morphology of Cyclorrhapha by way of taxonomic diagnoses, character identification and improvement in characters homologization.

**Electronic supplementary material:**

The online version of this article (doi:10.1007/s00436-014-4125-0) contains supplementary material, which is available to authorized users.

## Introduction

The cephaloskeleton of immature cyclorrhaphan Diptera is a structure providing data of great value in morphological studies. Cephaloskeleton details greatly reflect larval adaptation to the environment in which the larva lives (Ferrar 1987); however, they also reveal taxonomically and phylogenetically informative characters (e.g. Skidmore 1985; Ziegler 1998; Szpila 2010). Furthermore, phylogenetic tree resolution and its support increase when a holomorphology approach is applied, i.e. when both immature and adult characters are integrated in the analysis (Meier and Lim 2009). Thus, it is desirable to apply innovative microscopic methods to investigate cyclorrhaphan Diptera larvae in search of new phylogenetically informative characters. Larval morphology is traditionally studied using light microscopy. Examined structures are subsequently hand illustrated or photographed by microscope-mounted cameras. However, such visualization in a single plane neither conveys much 3D information nor the exact physical nature of interactions between characters of interest. Furthermore, in the case of fine structures, compound light microscopy studies are limited due to problems of resolution, illumination and the small depth of field not allowing for precise recognition of sclerites’ edges and interactions.

Innovative methods and new theoretical concepts have given new impetus to insect morphology studies (Friedrich et al. 2014). Nowadays application of scanning electron microscopy has caused a remarkable renaissance in larval morphology studies, especially species of either economic, medical or sanitary importance (e.g. Sukontason et al. 2008; Semelbauer and Kozánek 2012; Grzywacz et al. 2013; Szpila et al. 2014). However, while this technique is very useful for detailed imaging of the insect surface, it has no value for the investigation of internal structures.

Confocal laser scanning microscopy (CLSM) is a valuable tool for detailed studies of small, complex structures at high resolution close to the diffraction limit. CLSM depends on the degree of induced fluorescence of the examined samples (Lee et al. 2009). Fluorescence is aided either by staining with appropriate dyes or can be obtained using the autofluorescence properties of examined structures (Friedrich et al. 2014). By collecting fluorescence signals from different focal planes within a specimen, a fully three dimensional (3D) dataset can be acquired and used for visualization.

CLSM could have a profound impact on the quality of information provided over more traditional methods of imaging. CLSM application, e.g. in larval morphology studies, could provide hitherto overlooked data, particularly for small and complex sclerotized structures that can be difficult to observe and interpret by means of compound light microscopy (Schawaroch et al. 2005). Even though this technique allows for a very efficient examination of structural details, it has been neglected in an insect systematic and phylogenetic context (Lee et al. 2009; Friedrich et al. 2014). In morphological studies of Diptera, this technique has to date been applied in investigations of adult morphology, for example male genitalia (Klaus et al. 2003; Schawaroch et al. 2005; Klaus and Schawaroch 2006) and tarsomere details (Michels and Gorb 2012). None of the previous studies on cyclorrhaphan Diptera larval morphology applied CLSM methods. Furthermore, to our best knowledge the only CLSM application in Diptera immature stages investigation was a study of a mosquito larva (Zucker 2006).

Structures placed deep inside the specimen cannot be detected without clearing the specimen. Suitable agents for bleaching are, for example, methyl salicylate, KOH and Hoyer’s medium (e.g. Semelbauer and Kozánek 2012; Velásquez et al. 2013; Szpila et al. 2013, 2014). The aim of our study is to demonstrate the utility of confocal laser scanning microscopy for studying the morphological characters of cyclorrhaphan Diptera larvae by taking advantage of the autofluorescent properties of the cephaloskeleton. We investigated whether material cleared with KOH or Hoyer’s medium for compound light microscopy studies is suitable for CLSM studies. We compared results obtained for freshly prepared larvae with those obtained from slides stored in the collection of the Natural History Museum, London, UK.

## Material and methods

Material for the present study were both already prepared slides stored in the Natural History Museum (London, UK) collection and freshly prepared larvae of Calliphoridae (*Calliphora vicina* Robineau-Desvoidy and *Lucilia sericata* Meigen) and Muscidae (*Hydrotaea dentipes* (Fabricius), *Musca domestica* Linnaeus and *Muscina prolapsa* (Harris)). For the first and second instars, whole larvae were prepared, and for the third instars, anterior body ends were removed for subsequent preparation. Material was prepared according to two commonly used methods for compound light microscopy observations. Details of the cephaloskeleton placed deep inside the specimen have been revealed by means of clearing with potassium hydroxide or chloral hydrate (a key ingredient of Hoyer’s medium). A control sample was of larvae that were not cleared but directly embedded in water. Larvae were cleared with 10 % potassium hydroxide and subsequently either dehydrated through 80.0, 90.0, and 99.5 % ethanol (15 min in each) and slide mounted in Euparal (museum material) or, temporarily, slide mounted in water (fresh material). Immersion in KOH at room temperature in case of fresh material lasted 24 (first instar) or 48 h (second and third instars), yet no precise information on duration of KOH application was available for already prepared slides. Alternative clearing method involved direct mounting in Hoyer’s medium prepared according to Cielecka et al. (2009). Clearing with Hoyer’s medium was undertaken 3 weeks before CLSM examination to allow for better tissue penetration by the medium.

Samples were studied with a Nikon A1-Si Laser Scanning Confocal Microscope equipped with four different lasers with the following wavelengths: 405, 488, 561 and 640 nm. The autofluorescence signal was collected in four PMT channels with the following collection windows: 425–475 nm (blue), 500–550 nm (green), 570–620 nm (orange) and 685–725 nm (red). Structures were first imaged using a ×10 or ×20 dry objective lens (NA 0.3 or 0.7, respectively). Higher resolution data sets were then collected using a ×40 oil immersion lens (NA 1.3). Sequential images from a *z* stack were scanned and built up into maximum intensity projections (MIP) and subsequently 3D visualized.

## Results and discussion

Control samples, not cleared, were not found to be suitable for CLSM examination. Even if low autofluorescence had been emitted, we were unable to obtain data from deeper focal planes, i.e. cephaloskeleton, because of the lack of transparency of covering soft tissues and subsequent absorption of emitted light.

**Fig. 1:**
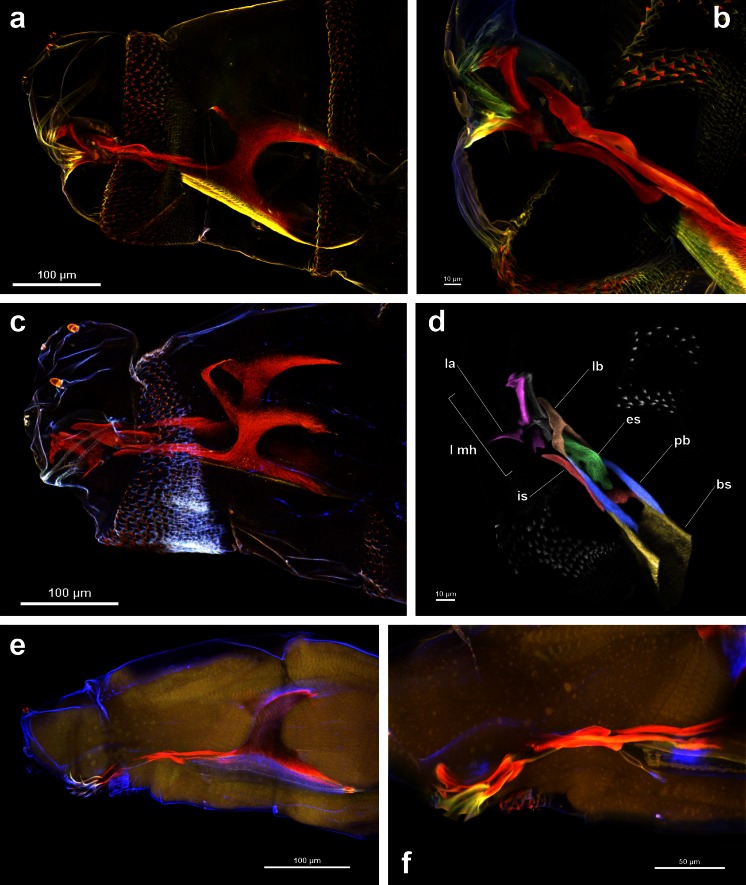
Anterior body end with the cephaloskeleton of the first instar larvae of *Calliphora vicina*, *Lucilia sericata* and *Hydrotaea dentipes*. **a** *L. sericata* cleared with KOH, embedded in Euparal [MIP of 28 optical sections collected with 4 lasers]. **b** *L. sericata* cleared with KOH, embedded in Euparal [MIP of 177 optical sections collected with 4 lasers]. **c** *C. vicina* cleared with KOH, embedded in water [MIP of 29 optical sections collected with 4 lasers]. **d** *L. sericata* cleared with KOH, embedded in Euparal [3D MIP of 177 optical sections same as Fig. 1b, yet rotated and collected with a 640-nm laser and visualized subsequently in B&W and with pseudo colours]. **e** *H. dentipes* cleared with Hoyer’s medium [MIP of 28 optical sections collected with 4 lasers]. **f** *H. dentipes* cleared with Hoyer’s medium [MIP of 226 optical sections collected with 4 lasers]

**Fig. 2:**
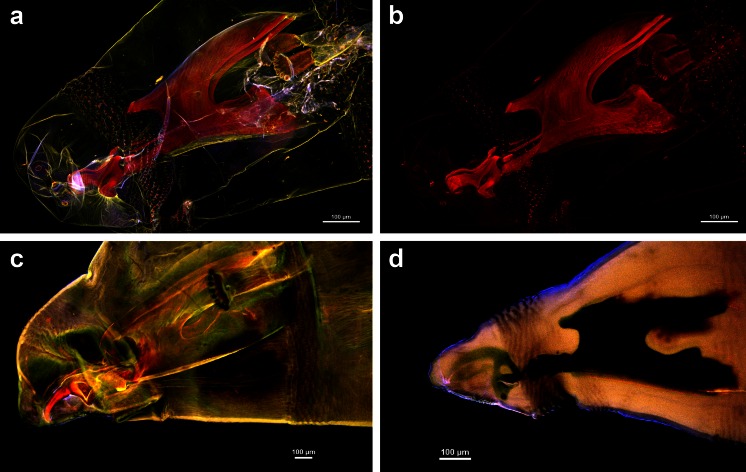
Anterior body end with cephaloskeleton of the second and third instar larvae of *Lucilia sericata* and *Muscina prolapsa*. **a** *L. sericata* cleared with KOH, embedded in Euparal [MIP of 27 optical sections collected with 4 lasers]. **b** *L. sericata* cleared with KOH, embedded in Euparal [MIP of 27 optical sections of the same larva as that imaged in Fig. 2b collected with a 640-nm laser]. **c** *L. sericata* cleared with KOH, embedded in Euparal [MIP of 12 optical sections collected with 4 lasers]. **d** *M. prolapsa* cleared with Hoyer’s medium [MIP of 11 optical sections collected with 4 lasers]

The cephaloskeleton is embedded within the anterior end of the larva, and it can be examined under a light microscope only when soft tissues are fully transparent. Thus, a clearing protocol is required to reveal its exact structure. Application of KOH or Hoyer’s medium are common methods in Diptera larval morphology studies (e.g. Semelbauer and Kozánek 2012; Szpila et al. 2014). Despite the fact that the key ingredient of Hoyer’s medium, chloral hydrate, makes soft tissues transparent and thus allows for detailed examination of cephaloskeleton details with a light microscope, this clearing technique is not applicable for CLSM studies. All three larval instars cleared with Hoyer’s medium were not suitable for CLSM studies because of low (Fig. 1e, f) or no (Fig. 2d) cephaloskeleton autofluorescence induction and, furthermore, absorption of emitted light by soft tissues.

Dark pigmentation of the cephaloskeleton hinders or even prevents the induction of autofluorescence. Potassium hydroxide is known to remove obstructing internal tissues and pigment (Schweiger et al. 2002), and it was used with success during our study in two ways. Treatment with 10 % KOH for 24–48 h (depending on larval instar and pigment intensity) led to maceration of soft tissues and reduction of cephaloskeleton pigmentation. Thereby, the induced high red autofluorescence was not absorbed by surrounding soft tissues and was enabled for detailed imaging (Figs. 1a–d, 2a–c). If larvae were cleared with 10 % KOH, the results obtained did not differ between slides stored in museum collection (Fig. 1a, b) or freshly prepared material (Fig. 1c). No differences were observed between slides mounted in water (Fig. 1c) and Euparal (Fig. 1a). However, it is recommended to use the latter medium, glycerine or Canada balsam (Friedrich et al. 2014), since high emission of heat by lasers during CLSM application causes water evaporation**.** Because KOH is commonly used as a clearing medium to examine the cephaloskeleton of Diptera larvae, it is possible to use microscopic slides prepared with this method for CLSM studies, since our results have shown that data obtained for freshly prepared and already slide-mounted larvae do not differ.

In the first instar of *L. sericata*, we discerned mouthhooks comprised of three sclerites not connected by a sclerotised patch (Fig. 1b, d). Such feature states have been reported in other blowflies, *C. vicina*, *Phormia regina* (Meigen) and *Lucilia illustris* (Meigen) (Szpila et al. 2008). Recent studies of the morphology of *L. sericata* Meigen and some other calyptrate larvae reported these three components connected with each other (Szpila and Villet 2011; Szpila et al. 2013). These differences result from the limits of compound light microscopy, which shows structures simultaneously from multiple focal planes, thus the sclerites’ edges and lines of demarcation can be blurred and confused by variation in the intensity of sclerotization (Schawaroch et al. 2005). CLSM better revealed the shapes and positions of individual structures and particularly their interconnections, as compared to standard light microscopy where fine structures, like mouthhooks sclerites, are often obscured.

An invaluable advantage of CLSM is 3D visualization and the possibility to rotate MIP about the *x*, *y* or *z* axis without any additional preparation (Fig. 1d and Online resource). This technique is especially useful for examination of already slide-mounted specimens (Lee et al. 2009). Thanks to 3D reconstruction, we discerned in particular the interaction of the epistomal sclerite with parastomal bars and the labrum in the first instar (Fig. 1d). The epistomal sclerite is equipped with four circular openings and fused anteriorly with the basal part of the labrum. The latter feature state was observed by Szpila et al. (2013); however, until recently, it has not been visualized exactly and Szpila et al. (2013) termed it the posterior expansion of the labrum, which is hump-shaped in Luciliinae. Grzywacz and Pape (2014) revealed that the presence of the epistomal sclerite in the first instar of Cyclorrhapha has been overlooked or confused by previous authors with only very few exceptions. The epistomal sclerite in successive instars has also been often overlooked or misidentified, for example as a labial sclerite (Arnaldos et al. 2014). Because the epistomal sclerite is often devoid of distinct sclerotization and present either in a form of a flat plaque (Velásquez et al. 2013) or a hump (Szpila et al. 2013), its occurrence can be overlooked even in the second and third instars. 3D visualization gives an invaluable ability to rotate MIP and detect all components of the cephaloskeleton.

In conclusion, CLSM and 3D reconstruction (Fig. 1d) are excellent techniques for visualizing fine, complex, autofluorescent structures of Dipteran larvae, if appropriate clearing techniques are first used. CLSM application can be an invaluable source of data for studies of larval morphology of Cyclorrhapha by way of taxonomic diagnoses, character identification and improvement in characters homologization. We recommend application of 10 % KOH for 24–48 h (depending on pigmentation intensity) and subsequent mounting in Euparal. CLSM could have a profound impact on the quality of information compared to more traditional methods of imaging. Cephaloskeleton details in the first instars of Cyclorrhapha have been recently recognized as valuable for taxonomic purposes (e.g., Szpila et al. 2013, 2014). However, their size and position relative to other sclerites obscure detailed descriptions. Thus, we recommend examination of cephaloskeleton of the first instar of other cyclorrhaphan Diptera with CLSM, because as it has been show herein, this method better reveals the shapes and positions of individual structures and particularly their interconnections, as compared to standard light microscopy. In the successive larval instars, despite relatively bigger size of the cephaloskeleton sclerites, observation of borders between closely apposed sclerites (see Grzywacz and Pape 2014) can still cause problems. Thus, also for the second and third instars, CLSM and especially 3D reconstruction are highly recommended for better understanding of cephaloskeleton details.

## Electronic supplementary material

Below is the link to the electronic supplementary material.

ESM 1 (MPG 5382 kb)

## Abbreviations

bs: Basal sclerite
es: Epistomal sclerite
is: Intermediate sclerite
l mh: Left mouthhook
la: Lateral arm
lb: Labrum
pb: Parastomal bar

## References

[CR1] Arnaldos MI, Ubero-Pascal N, García R, Carles-Tolrá M, Presa JJ, García MD (2014) The first report of *Telomerina flavipes* (Meigen, 1830) (Diptera, Sphaeroceridae) in a forensic case, with redescription of its pupa. Forensic Sci Int 242:e22–e30 10.1016/j.forsciint.2014.07.023 25128390

[CR2] Cielecka D, Salamatin R, Garbacewicz A (2009). Usage of Hoyer’s medium for diagnostic and morphological studies of some parasites. Wiad Parazytol.

[CR3] Ferrar P (1987) A guide to the breeding habits and immature stages of Diptera Cyclorrhapha. Entomonograph Vol. 8

[CR4] Friedrich F, Matsumura Y, Pohl H, Bai M, Hörnschemeyer T, Beutel RG (2014). Insect morphology in the age of phylogenomics: innovative techniques and its future role in systematics. Entomol Sci.

[CR5] Grzywacz A, Pape T (2014). Larval morphology of *Atherigona orientalis* (Schiner) (Diptera:Muscidae)—a species of sanitary and forensic importance. Acta Trop.

[CR6] Grzywacz A, Pape T, Hudson WG, Gomez S (2013). Morphology of immature stages of *Atherigona reversura* (Diptera: Muscidae), with notes on the recent invasion of North America. J Nat Hist.

[CR7] Klaus AV, Schawaroch V (2006). Novel methodology utilizing confocal laser scanning microscopy for systematic analysis in arthropods (Insecta). Integr Comp Biol.

[CR8] Klaus AV, Kulasekera VL, Schawaroch V (2003). Three‐dimensional visualization of insect morphology using confocal laser scanning microscopy. J Microsc.

[CR9] Lee S, Brown RL, Monroe W (2009). Use of confocal laser scanning microscopy in systematics of insects with a comparison of fluorescence from different stains. Syst Entomol.

[CR10] Meier R, Lim GS (2009). Conflict, convergent evolution, and the relative importance of immature and adult characters in endopterygote phylogenetics. Ann Rev Entomol.

[CR11] Michels J, Gorb SN (2012). Detailed three‐dimensional visualization of resilin in the exoskeleton of arthropods using confocal laser scanning microscopy. J Microsc.

[CR12] Schawaroch V, Grimaldi D, Klaus AV (2005). Focusing on morphology: applications and implications of confocal laser scanning microscopy (Diptera: Campichoetidae, Camillidae, Drosophilidae). Proc Entomol Soc Wash.

[CR13] Schweiger PF, Rouhier H, Söderström B (2002). Visualisation of ectomycorrhizal rhizomorph structure using laser scanning confocal microscopy. Mycol Res.

[CR14] Semelbauer M, Kozánek M (2012). Morphology of preimaginal stages of *Lauxania* and *Calliopum* (Diptera: Lauxaniidae). Zootaxa.

[CR15] Skidmore P (1985). The biology of the Muscidae of the world. Ser Entomol.

[CR16] Sukontason KL, Sribanditmongkol P, Chaiwong T, Vogtsberger RC, Piangjai S, Sukontason K (2008). Morphology of immature stages of *Hemipyrellia ligurriens* (Wiedemann) (Diptera: Calliphoridae) for use in forensic entomology applications. Parasitol Res.

[CR17] Szpila K (2010) The first instar of European Miltogramminae (Diptera: Sarcophagidae). Nicolaus Copernicus University Press

[CR18] Szpila K, Villet M (2011). Morphology and identification of first instar larvae of African blowflies (Diptera: Calliphoridae) commonly of forensic importance. J Med Entomol.

[CR19] Szpila K, Pape T, Rusinek A (2008). Morphology of the first instar of *Calliphora vicina*, *Phormia regina* and *Lucilia illustris* (Diptera, Calliphoridae). Med Vet Entomol.

[CR20] Szpila K, Hall MJR, Pape T, Grzywacz A (2013). Morphology and identification of first instars of the European and Mediterranean blowflies of forensic importance. Part II. Luciliinae. Med Vet Entomol.

[CR21] Szpila K, Hall MJR, Wardhana AH, Pape T (2014). Morphology of the first instar larva of obligatory traumatic myiasis agents (Diptera: Calliphoridae, Sarcophagidae). Parasitol Res.

[CR22] Velásquez Y, Ivorra T, Grzywacz A, Martínez-Sánchez A, Magaña C, García-Rojo A, Rojo S (2013). Larval morphology, development and forensic importance of *Synthesiomyia nudiseta* (Diptera: Muscidae) in Europe: a rare species or just overlooked?. Bull Entomol Res.

[CR23] Ziegler J (1998) The morphology of the puparia and of the cephalo-pharyngeal skeleton of mature larvae of tachinid flies (Diptera, Tachinidae) and their phylogenetic significance. Stud Dipter Suppl 3 [in German with English abstract]

[CR24] Zucker RM (2006). Whole insect and mammalian embryo imaging with confocal microscopy: morphology and apoptosis. Cytom A.

